# Transient Microstructure Evolutions and Local Properties of Dual-Phase 980 MPa Grade Steel Via Friction Stir Spot Processing †

**DOI:** 10.3390/ma13194406

**Published:** 2020-10-02

**Authors:** Koichi Taniguchi, Yong Chae Lim, Alexis Flores-Betancourt, Zhili Feng

**Affiliations:** 1 Steel Research Laboratory, JFE Steel Corporation, Chiba 260-0835, Japan; ko-taniguchi@jfe-steel.co.jp; 2 Materials Science and Technology Division, Oak Ridge National Laboratory, Oak Ridge, TN 37831, USA; limy@ornl.gov (Y.C.L.); floresbetana@ornl.gov (A.F.-B.)

**Keywords:** friction stir processing, advanced high-strength steel, characterization, microstructures

## Abstract

Friction stir processing is a novel solid-state process to modify microstructures and their properties by intense, localized plastic deformation. However, little research has been reported for microstructure evolutions of advanced high-strength steels during the process. The present work focuses on the study of transient microstructure changes and local mechanical properties for friction stir spot processed dual-phase (DP) 980 MPa grade steel (DP980) under different peak temperatures. A pinless silicon nitride ceramic tool was used to produce relatively simple material deformation and flow near the tool. Friction stir spot processed steel samples were characterized by optical and electron microscopies. Furthermore, Vickers microhardness and nano-indentation measurements were used to study local mechanical properties for correlation with microstructures. A swallow layer of refined grains (<0.6 µm) was obtained with a low peak temperature (under 400 °C), whereas higher peak temperatures (>Ac_1_) led to a change in grain size with different microstructures (fine-grained DP or martensite). Electron back-scattered diffraction characterizations revealed a large deformation in the as-received microstructures (mixture of ferrite and tempered martensite) induced by friction stir spot processing, leading to recrystallization and grain refinement around the stirred zone. Also, nano-indentation measurements showed a higher hardness than the hardness of the as-received DP980. Friction stir processing with different process conditions effectively changed microstructures and local mechanical properties.

## 1. Introduction

Advanced high-strength steel (AHSS) with a tensile strength of 780 MPa or higher has been used in automotive industries to reduce the car body weight for higher fuel efficiency [1,2,3]. Resistance spot welding has been widely used for AHSS to integrate into vehicle body-in-white structures, but some challenges remain to obtaining high weld qualities and mechanical joint properties. For example, segregation of alloying elements such as phosphorous and sulfur into the grain boundary during solidification inevitably deteriorated the toughness of the martensite, leading to low cross-tension strength [4,5]. Therefore, developing suitable welding techniques is essential for transportation industries, and developing new AHSS is essential for steel manufacturers. To avoid melting and resolidification issues from the fusion welding process, friction stir-based welding (FSbW) technologies [6] have been developed as a solid-state material process and joining technique. Examples of such techniques include friction stir welding (FSW) [7], friction stir spot welding (FSSW) [8], friction stir processing (FSP) [7], and variations of friction stir riveting [9]. The applicability of FSbW on AHSS has been studied for the past two decades. Santela et al. reported that FSSW of 780 MPa grade AHSS can achieve high lap shear strength over the industrial standard [10], and Matsushita et al. illustrated the FSW parameter window of 1180 MPa grade AHSS, which achieved complete consolidation without damaging the tool [11]. Friction bit joining was also successfully conducted in dissimilar welding of 980 MPa AHSS and an Al alloy [12]. These works prove the applicability of FSbW technologies on AHSS.

However, some challenges still exist in spreading the use of FSbW in industries. A tool shape has been investigated to avoid tool wear and deterioration of joint strength caused by a “hook” unbonded interface and keyhole. Recent research on FSSW of an Al alloy revealed that a pinless tool can form a good joint without the hook interface and keyholes [13,14,15]. Mousavizade et al. applied pinless FSSW on a mild steel sheet with a projection-backing anvil and proved that the technique provided high tensile strength because of the absence of keyholes [16]. Although the pinless tool approach during FSSW is a promising way to achieve a high-quality joint of AHSS, the published research on this subject is still limited.

Another challenge is the lack of understanding of the relationship between microstructure evolution and local mechanical properties during the process. Microstructure evolution during the process is complex because several factors, such as inclusions, affect dislocation, texture, and phase transformation [17]. Aktrer et al. intensely evaluated friction stir processed DP600 using EBSD and revealed grain refinement from 2.83 to 0.79 µm in the stir zone (SZ) due to phase transformation and dynamic recrystallization [18]. Strengthening factors in friction stir processed (FSPed) microstructures have been identified in other steel materials. Baker et al. evaluated the grain boundary strengthening factor in FSWed oxide dispersion strengthened (ODS) steel based on the Hall-Petch equation and found that the coarsening oxide particle and grain size reduced yielding stress for FSWed ODS steel [19]. Li et al. found that the lower hardening capacity (the ratio of the ultimate tensile strength to the yield strength) of FSPed reduced-activation ferritic/martensite (RAFM) steel due to grain refinement strengthening effect based on the Hall-Petch relationship and dislocation strengthening effect [20]. Hua et al. explained that the hardenability of FSWed 12Cr steel was consistent with grain refinement, carbon content, and the existence of dispersoids [21]. Also, they observed grain refinement in the SZ through EBSD. Mazaheri et al. successfully calculated yield stress of DP980 manufactured by a thermomechanical process based on the accumulation of each strengthening effect [22]. Therefore, investigation of each strengthening effect for various thermomechanical process conditions in steels is essential for understanding the local and global mechanical properties with respect to various microstructures. However, few reports still exist about transient microstructures and local mechanical properties.

Hardness testing is traditional and essential to assess local properties. In recent works, Pavlina et al. summarized the correlation of yield strength and tensile strength with Vickers microhardness of various materials [23]. A nano-indentation technique was employed to evaluate local strength. Baltazar Hernandez et al. conducted nano-indentation tests on a resistance spot weld of DP980 and reported a softened area was contributed with both softening ferrite and tempered martensite [24,25]. Local mechanical properties have been validated through miniature mechanical testing using simulated samples [26]. However, little effort has been made on the local microstructural characteristics of FSWed AHSS.

Understanding in transient microstructures and the local characteristics has another benefit for automotive and steel industries. Recent work has revealed that higher mechanical properties, including toughness, can be obtained by refined microstructures by processing under Ac_1_ temperature [27]. Understanding metalogical phenomena in low temperatures is essential to maximize FSPed or FSWed AHSS.

For these reasons, this work encompasses experimental efforts to investigate transient microstructures and their local properties under different friction stir spot processing (FSSP) conditions. The authors conducted FSSP on 980 MPa grade AHSS using a pinless tool to evaluate the material deformation near the contact area of the tool and the material. The microstructures were characterized by optical microscopy (OM), scanning electron microscopy (SEM), and EBSD. Vickers microhardness and nano-indentation measurements were conducted to study local mechanical properties. Transient changes in microstructures during FSSP for AHSS are discussed regarding the temperature and deformation state. Finally, strengthening effects were studied for different FSSP conditioned steel samples.

## 2. Materials and Methods

### 2.1. Materials

**Figure 1:**
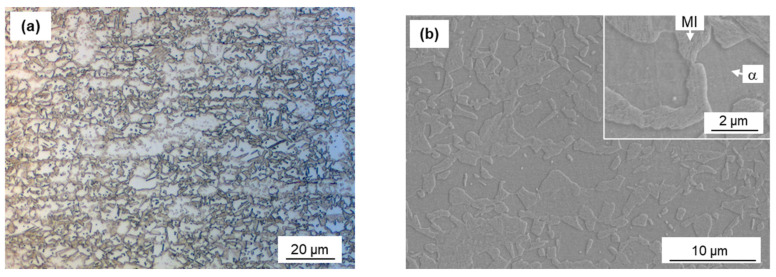
(**a**) Optical micrograph image of the base metal (BM) and (**b**) magnified SEM of the BM, showing mixture of ferrite (α) and martensite island (MI).

A 1.2 mm thick cold rolled dual-phase (DP) 980 MPa grade steel sheet (called “DP980” in this study), which consisted of ferrite and martensite, was used for experiment. The chemical composition of the DP980 was 0.12C-0.9Si-2.1Mn-0.01P-0.002S (mass %). Yield stress and tensile stress were 690 and 1060 MPa, respectively. The Ac_1_, Ac_3_, and M_s_ transformation temperatures of the base metal (BM) with a given chemical composition were predicted to be 721 °C, 870 °C, and 403 °C, respectively [28]. Figure 1 shows images of OM and magnified SEM micrographs showing the microstructures of the BM. The microstructures exhibited a typical structure for DP AHSS with ferrite (α) and a martensite island (MI) as shown in Figure 1b. The volume fraction of martensite (f_M_) was approximately measured to be 0.6, whereas the volume fraction of ferrite was measured as 0.4, based on Figure 1. Dimensions of test coupons were 100 mm long and 30 mm wide. Prior to experimentation, all coupons were cleaned with acetone to remove any dirt or oil on the sheet surface.

### 2.2. Friction Stir Spot Processing

**Figure 2:**
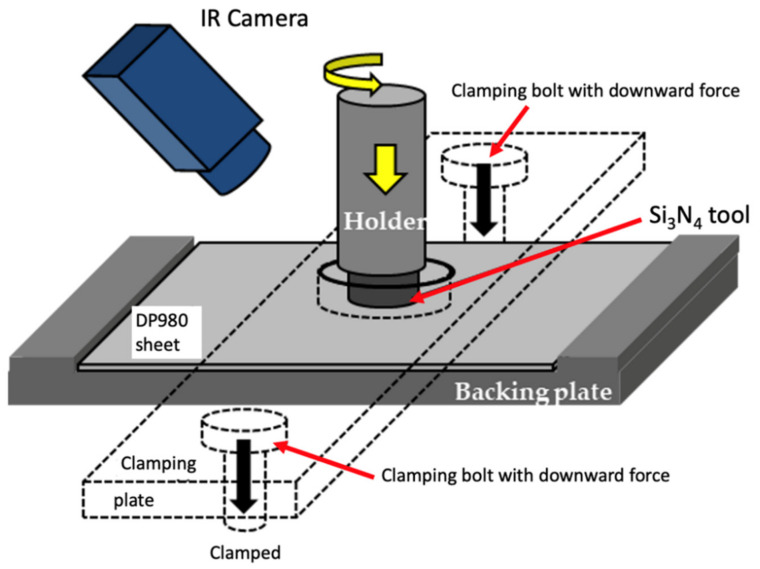
Schematic of friction stir spot processing (FSSP) experimental setup showing IR camera, tool holder, Si_3_N_4_ tool, DP980 steel sheet, clamping, and backing plates.

To avoid complex and heterogeneous material flow during FSSP, a flat tool (i.e., without a pin) was employed. The tool material was a silicon nitride (Si_3_N_4_) and the shoulder diameter was 14 mm. The experimental setup for FSSP is illustrated in Figure 2. To prevent any undesired motion during FSSP, a DP980 sheet was mounted on a steel backing plate and tightly clamped by another steel clamping plate with two steel bolts on both sides. Initially, various FSSP conditions were conducted to correlate peak temperatures at the tool edge and microstructures. Based on the preliminary experiment and examination, three FSSP parameters were down-selected for more detailed investigation as summarized in Table 1. Dwell time is the actual duration since the force reaches the plunging force until the force starts to decrease. In this manuscript, the samples processed with conditions A, B, and C are called sample A, sample B, and sample C.

During FSSP, the peak temperature on the processed surface was recorded by a FLIR SC655 infrared (IR) (FLIR, Wilsonville, OR, USA) thermal imaging camera with a frequency of 50 Hz acquisition rate. The IR camera was positioned on perspective view and aimed at the tool edge and steel substate. Based on the preliminary experiment, the temperature range for the IR camera was set from 0 °C to 650 °C for condition A and from 100 °C to 1200 °C for conditions B and C.

### 2.3. Metallographic Sample Preparation

All samples were cut at the center of the processed area and mounted in epoxy for cross-sectional observation. For OM and SEM characterization, the samples were grinded and polished through 1 µm diamond paste, then etched with 2% Nital reagent. For EBSD characterization, the samples were further polished through 0.02 µm colloidal silica.

For nano-indentation, the samples were finish polished with 0.5 µm diamond solution before measurement to obtain a fine surface. After indentation test, the samples were etched with 2% Nital for post-SEM characterization to correlate the microstructures and local mechanical properties.

### 2.4. Characterizations of Microstructures

The microstructures of friction stir spot processed surface was characterized using a Zeiss Axiocam MRc5 optical microscope (Carl Zeiss Microscopy, LLC, White Plains, NY, USA), Hitachi S4800 field emission SEM (Hitachi High-Tech, Tokyo, Japan) and a JEOL 6500F (JEOL, Peabody, MA, USA) or TESCAN MIRA3 XMH Schottky field emission SEM (Tescan, Brno-Kohoutovice, Czech Republic) equipped with an EBSD. Accelerating voltage was 20 kV for all measurement. Step size was set from 0.1 µm to 0.3 µm. Average confidence indexes (CIs) were from 0.12 to 0.25. Misorientations of 5 to 15° between neighboring points were defined as low-angle grain boundaries (LAGBs), whereas misorientations over 15° were defined as high-angle grain boundaries (HAGBs). Substructure boundaries (SSBs) were defined when the misorientation angle was lower than 5°. The f_M_ was measured using the segmentation method on optical microscopy or SEM images.

### 2.5. Vickers Microhardness and Nano-Indentation

Two types of Vickers microhardness tests were performed using a Leco microhardness tester (LM 100AT) (Leco, Saint Joseph, MI, USA). Macroscopic hardness mapping was conducted through an evaluation under a 200 gf load and 10 s dwell time with 100 μm steps in both directions. Another set of Vickers microhardness tests were conducted under a 25 gf load and 10 s dwell time with a 15 µm pitch from the top surface through thickness direction, which means nominal direction of the surface, to assess the hardness change near the top surface.

Nano-indentation was performed to study local mechanical properties for different microstructures after FSSP. A Hysitron triboindenter Ti-900 (Bruker, Eden Prairie, MN, USA) equipped with a Berkovich diamond tip and a scanning probe microprobe for imaging was employed. The Berkovich diamond tip was calibrated by using a reference specimen of fused silica. Specimens were nano-indented for the fine polished surface with a load of 3000 µN. A total of 840 indentations were performed in an area of 100 µm and 200 µm with a pitch of 5 µm.

## 3. Results and Discussion

### 3.1. Thermal History During Friction Stir Spot Processing 

**Figure 3:**
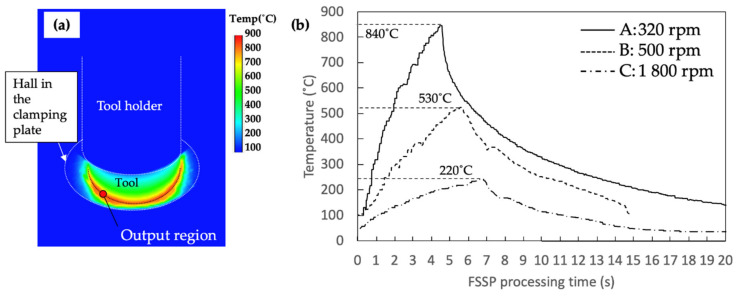
Thermal history measured by IR camera: (**a**) snapshot image of temperature distribution at 4.7 s with process condition C (i.e., the 1800 rpm case); and (**b**) time–temperature curves for each condition measured by IR camera.

Directly measuring the peak temperature underneath the tool was difficult because of the interaction between the tool and top surface of the material during FSSP. In this study, the temperature profile at the near edge of the silicon nitride tool on the steel surface was measured by an IR camera in Figure 3a, because emissivity of the silicon nitride was close to 1 within a wide range of temperatures [29]. Figure 3a shows an example of snapshot image during FSSP with condition C (i.e., the 1800 rpm case). The peak temperature on the friction stir spot processed surface can be found at the Si_3_N_4_ tool edge. The time-temperature curves of each sample captured by the IR camera are given in Figure 3b. The measured peak temperatures in condition A, B, and C were 220 °C, 530 °C, and 840 °C, respectively. However, Yang et al. found that the peak temperature in the friction stir processed zone can be higher than at the edge [15]. Therefore, the actual temperature at different locations should be further correlated from the microstructure and hardness distribution.

### 3.2. Characterization of Microstructures and Vickers Microhardness Measurement

**Figure 4:**
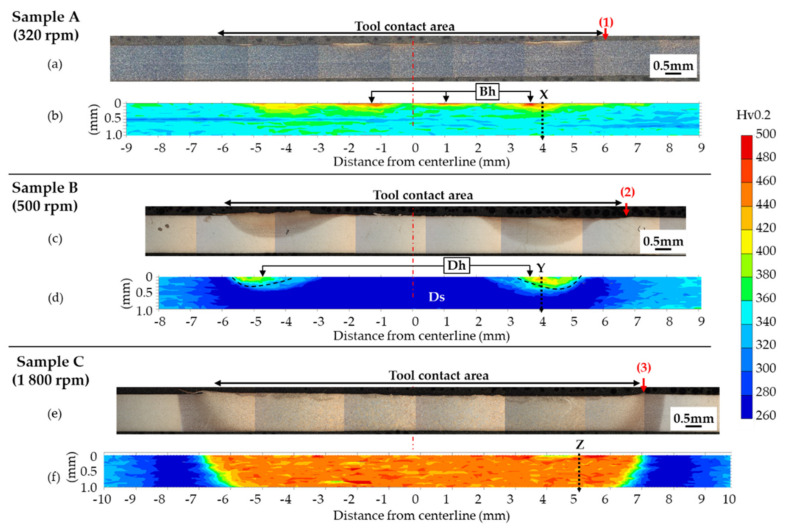
Cross-sectional optical microscopy (OM) image of macrostructures and a mapping of Vickers microhardness distribution: (**a**), (**b**), (**c**), (**d**), and (**e**), (**f**) are OM images and hardness distributions of sample A, B, and C, respectively. The tool edge for each sample was marked as “1,” “2,” and “3.” Location “Bh” and “Dh” in sample A and sample B, respectively, were the hardened areas. Location “Ds” was the softened area in sample B. Dashed line “X,” “Y,” and “Z” were selected for detailed characterization in the following section.

Figure 4 shows a macroscopic image of the cross-section and corresponding Vickers microhardness mapping of each sample (i.e., low, mid, high peak temperature at the edge of the tool). As shown in Figure 4a,b, localized surface modification was found on the steel sample. That is, islands of nonuniform microstructural change and a hardened area, where the hardness was over 328 Hv (green or yellow), were found near the surface marked as “Bh” in Figure 4b. Next, as seen in Figure 4c,d, sample B (i.e., mid peak temperature at the edge of the tool) showed two modified areas near the edge of the tool with distinctive microstructural changes, which correlated to a hardened area labeled as “Dh” in Figure 4d. The Vickers microhardness ranged from 360 to 420 Hv at two distinctive areas. The rest of the areas (dark blue color in Vickers microhardness map) were found to be softened and ranged from 260 to 300 Hv in Vickers microhardness as indicated “Ds” in Figure 4d. Finally, sample C (i.e., higher peak temperature at the edge of the tool) had uniform microstructural change under the entire tool contact area as shown in Figure 4e. This area was the most hardened area under the tool; the measured Vickers microhardness ranged from 420 to 500 Hv in Figure 4f. Additionally, gradual microhardness changes through the lateral direction were found for samples B and C.

**Figure 5:**
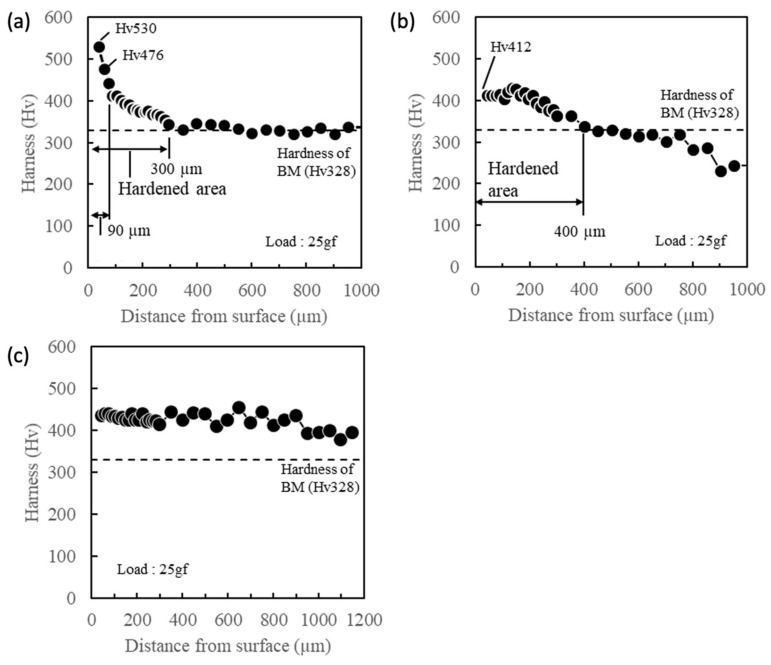
Vickers microhardness distribution through the thickness direction (**a**) sample A for low peak temperature; (**b**) sample B for mid peak temperature; and (**c**) sample C for high peak temperature.

The most hardened zone in each sample was further evaluated because they were considered to be affected by the largest thermo-mechanical process during FSSP. For this reason, dashed line “X” and “Y” in Figure 4b,d were chosen for sample A and sample B, respectively. In sample C, dashed line “Z” in Figure 4f was selected as one of the areas hardened through the area near the tool edge. Figure 5a,b,c show Vickers microhardness distribution along the thickness direction from the top surface to bottom surface for each sample in dashed line “X,” “Y,” and “Z” in Figure 4b,d,f. As seen in Figure 5a, measured Vickers microhardness was higher than 400 Hv within a 90 µm distance from the top surface. Also, up to 300 µm away from the top surface, measured microhardness was slightly higher than the base metal (= 328 Hv). Next, in Figure 5b, 400 µm from top surface was hardened to 400 Hv in sample B and the hardness gradually decreased with an increasing distance from the top surface. Finally, the microhardness was higher than 400 Hv through the thickness direction in sample C.

**Figure 6:**
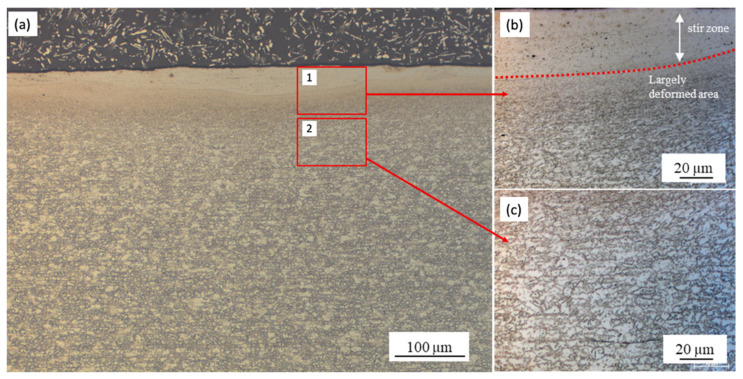
(**a**) OM image near the top surface of a sample with condition A (Location “X” in Figure 4b) showing different states of microstructures; (**b**) magnified optical images at location “1” showing SZ (red dotted line) and largely deformed area near top surface; and (**c**) magnified optical image at location “2” showing mixture of ferrite and martensite microstructures.

**Figure 7:**
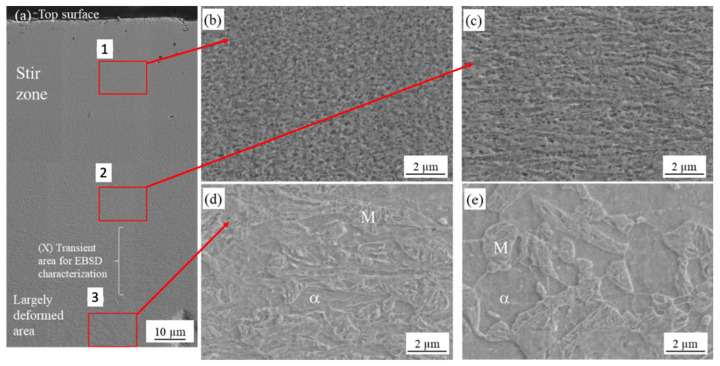
SEM micrographs of the cross-section near the top surface of sample A: (**a**) low-magnified cross-sectional SEM image near the top surface. Red squares mark locations from “1” to “3” are for SZ, transient zone, and deformed zone, respectively; (**b**) magnified SEM image at the SZ indicated as “1,” which consists of very fine-grained microstructures under 0.6 µm; (**c**) magnified SEM image at transient zone marked as “2,” consisting of small and deformed microstructures; (**d**) magnified SEM image at deformed area marked as “3” where microstructures are largely deformed; and (**e**) area at a distance of 200 µm away from the top surface, whose microstructures are similar to the BM.

**Figure 8:**
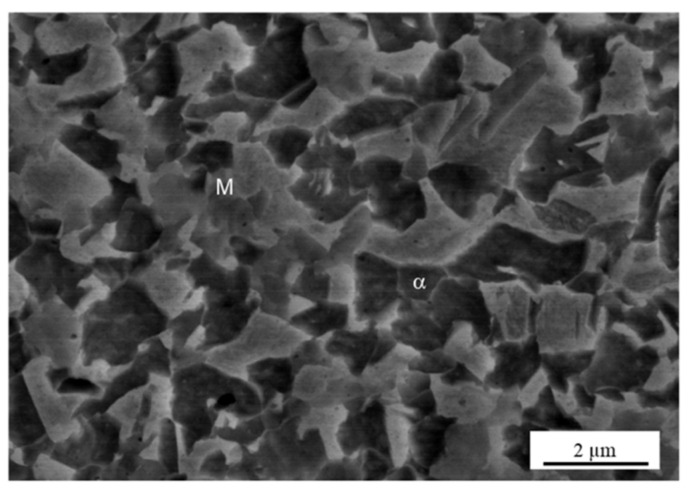
SEM micrograph of microstructures (mixture of refined ferrite (α) and martensite (M)) at the top surface for sample B top surface.

**Figure 9:**
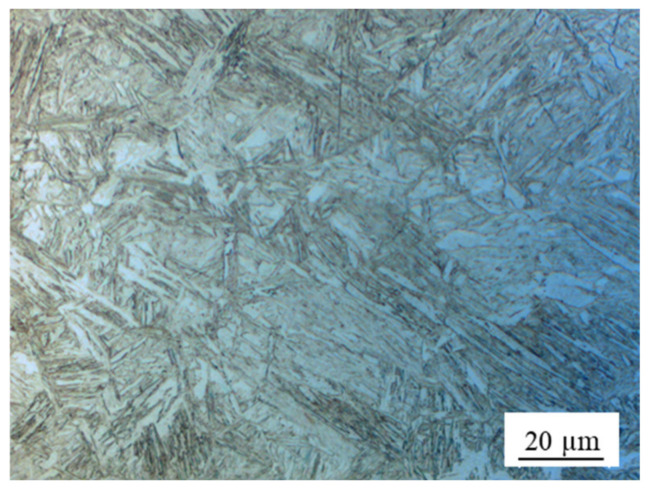
Optical microcopy image of microstructure (fully martensite) at the top surface for sample C.

Figure 6 presents macroscopic cross-section and magnified optical images of the top surface of sample A. Figure 6b depicts the SZ at the top surface (location “1” in Figure 6a) and the locally deformed area. Figure 6c shows a magnified OM image of mixture of ferrite and martensitic microstructure at location “2” in Figure 6a. Figure 7a shows a magnified SEM micrograph of sample A with different locations from the top surface as previously shown in Figure 6a. Further magnified SEM for each location is shown in Figure 7b,c,d. As seen in Figure 7b, the SZ consisted of very fine grains at least under 0.6 µm measured by EBSD in the next section. Around the SZ, largely deformed microstructures were observed (Figure 7c) and deformation looked smaller with an increasing distance from the top surface (Figure 7d). Microstructures at the 200 µm distance from the top surface were similar to the BM (mixture of ferrite and martensite) as shown in Figure 7e. The f_M_ at each location was approximately measured to be 0.6. In a similar manner, Figure 8 shows an SEM micrograph of microstructures near the top surface of sample B. Microstructures consisted of DPs (i.e., a mixture of ferrite and martensite) without significant deformation and f_M_ was measured as 0.58. The microstructure on the top surface for condition C essentially was fully martensite, where f_M_ was 1.0 as seen in Figure 9.

From microstructure characterization for each sample condition, actual peak temperature generated for the location where the tool and the steel sample directly contacted during FSSP was estimated. For sample A, the peak temperature was estimated to be at least under 400 °C because no softening was observed. Similarly, Hernandez et al. found no softening area under 400 °C through a spot welding on the same steel sheet [25]. No change in the f_M_ also proves that the peak temperature was adequately lower than the Ac_1_ temperature. Next, the peak temperature for sample B was estimated between Ac_1_ and Ac_3_ (i.e., intercritical) mainly because of the refined DP microstructure shown in Figure 8. The difference between Ac_1_ (721 °C) and the measured temperature (530 °C) can be explained by two potential reasons. First, estimated peak temperature in the friction stir spot processed Al alloy was 100 °C higher because of the higher thermal conductivity of Al [15]. Steel has lower thermal conductivity than the Al, so estimated peak temperature for steel can be higher than the measured temperature by the IR camera. Second, the microstructure characterized area was 3 mm away from the tool edge in Figure 4c. Finally, the peak temperature in sample C was over Ac_3_ because the measured temperature was around Ac_3_ and the microstructures were essentially martensite structures.

The difference of the island-shape microstructural change was consistent with the transient change in the dominant effect on microstructures near the top surface. The nonuniform hardness distribution and deformed state in Figure 6 suggest the material contacting with the tool was locally deformed, but the wide deformation was limited because of the low material flow at the low temperature. In this case, the microstructural change was largely affected by mechanical processing only from the tool. Increasing tool rotation speeds concentratedly heated the top surface near the tool edge, and then phase transformation became dominant because of heat conduction to the surrounding areas.

### 3.3. Characterization of Microstructures

**Figure 10:**
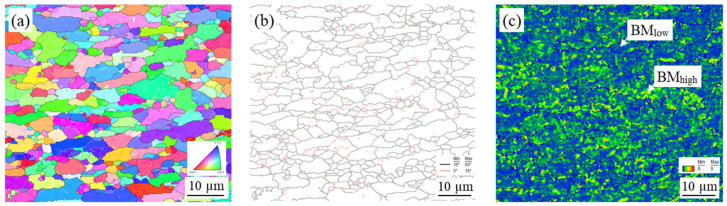
EBSD characterization of the BM: (**a**) inverse pole figure; (**b**) grain boundary; and (**c**) KAM.

**Figure 11:**
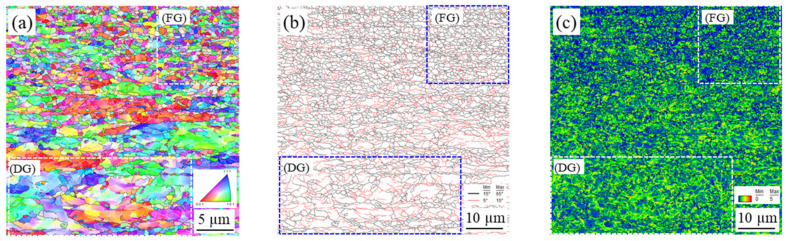
EBSD characterization at the transient area from fine-grained (FG) to deformed-grained (DG) near the top surface of sample A: (**a**) inverse pole figure; (**b**) grain boundary; and (**c**) kernel average misorientation (KAM).

**Figure 12:**
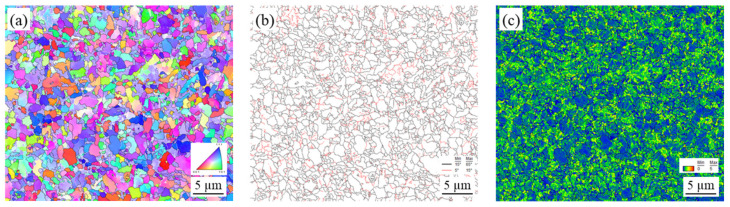
EBSD characterization at the representative FG area near the top surface of sample B: (**a**) inverse pole figure; (**b**) grain boundary; and (**c**) KAM.

**Figure 13:**
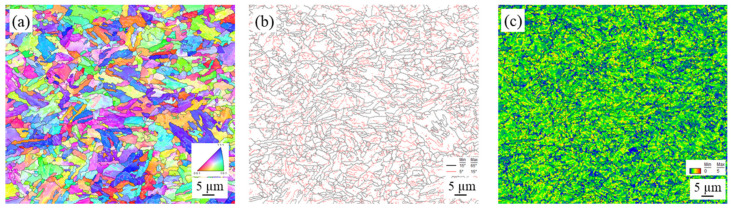
EBSD characterization at the representative FG area near the top surface of sample C: (**a**) inverse pole figure; (**b**) grain boundary; and (**c**) KAM.

EBSD was used to study grain size and retained strain in the microstructures under different thermal and mechanical conditions induced by FSSP. Figure 10, Figure 11, Figure 12, and Figure 13 show the inverse pole figure, grain boundary, and kernel average misorientation (KAM) of the BM and samples A, B, and C, respectively. The KAM map of a representative area in the BM is shown in Figure 10c, indicating a low area (location BM_low_) and high area (location BM_high_). For sample A, the transient zone observed in Figure 7a was evaluated. The grain size transiently changed from the fine-grained (FG) area to the deformed-grained (DG) area in Figure 11. Fewer sub-grains were found and KAMs were lower at the FG area than at the DG area as shown in Figure 11c. Next, grains in sample B showed little distortion but random direction to neighboring grains as shown in Figure 12. The KAM map in Figure 12c consisted of a low and high area. Finally, complex structures were found in sample C and the KAMs in Figure 13 in all areas were higher than the other samples.

**Figure 14:**
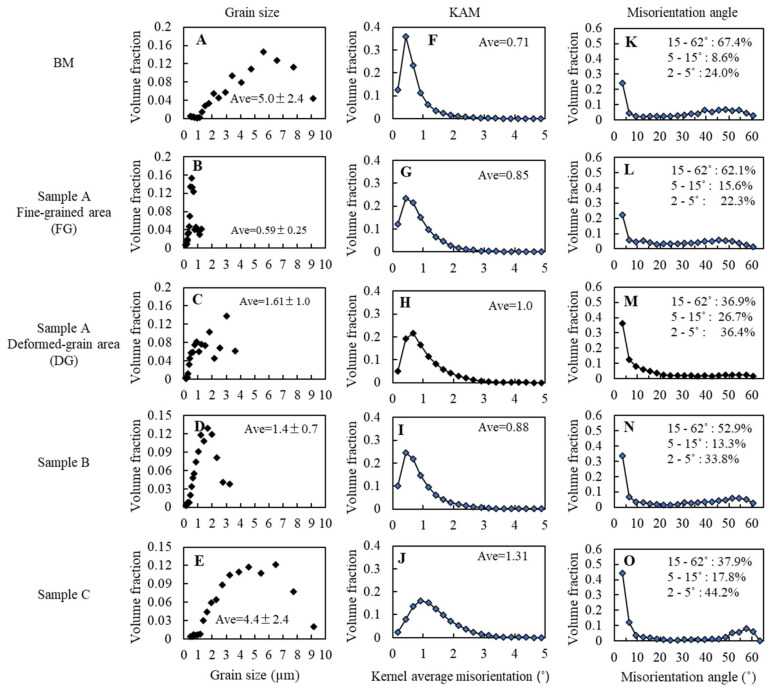
Comparison of grain size, KAM, and misorientation angle characterized by EBSD: (**A**), (**F**), and (**K**) are for the BM; (**B**), (**G**), and (**L**) are for the FG zone of sample A; (**C**), (**H**), and (**M**) are for the DG zone of sample A; (**D**), (**I**), and (**N**) are for sample B; and (**E**), (**J**), and (**O**) are for sample C.

To compare the characteristics of each sample, the distributions of grain sizes, KAMs, and misorientation angles are summarized in Figure 14. Grain sizes of the BM, FG, and DG for sample A were 5.0 ± 2.4 µm, 0.59 ± 0.25 µm, and 1.61 ± 1.0 µm, respectively. Grain sizes at the FG area near top surface of sample B and sample C were 1.4 ± 1.0 µm and 4.4 ± 2.4 µm, respectively. Average KAM angles for the BM, FG in sample A, and DG in sample A, sample B, and sample C were 0.71°, 0.85°, 1.0°, 0.88°, and 1.31° as found in Figure 14F–J respectively. HAGBs (15–65°), LAGBs (5–15°), and SSBs (<5°) of the BM were 67.4%, 8.6%, and 24%, respectively, as shown in Figure 14K. The LAGB fraction of DG in sample A was 26.7%, which was higher than other areas. The SSB fraction of FG in sample A was close to the BM, although the fraction was higher (over 30%) at other locations or samples.

**Figure 15:**
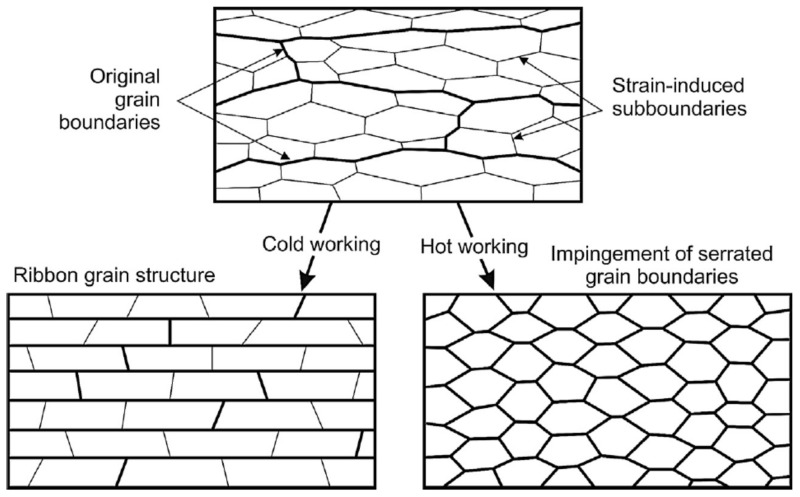
Schematic of ultra-fine grain (UFG) development during unidirectional plastic working to large strains. Directional ribbon grain structures formed by a grain subdivision process at room temperature or through the geometric dynamic recrystallization at elevated temperatures [30].

The results suggest that grain refinement can occur in the initial stage of processing under A_c1_ and the grain size can be smaller than 0.6 µm as shown in Figure 7 and Figure 14. Grain refinement can be obtained as a result of severe strain during FSSP. Under severe plastic deformation in a low temperature, high dislocation density substructures can form and subsequently develop into new grains as illustrated in Figure 15 [30]. Further deformation can result in the development of lamellar microstructures, where both the original as well as the strain-induced HABs rotate into the rolling plane as shown by Sakai et al. [30]. The HAB spacing decreased with strain, approaching the cell and sub-grain size at large strains. This mechanism resembles that of geometric dynamic recrystallization.

The results of sample A support the mentioned mechanisms. Microstructures were transiently changed from deformed DP to lamellar-like structures and finally very fine grains under 0.59 µm. The lamellar-like structures shown in Figure 7c were similar to a ribbon grain structure after severe plastic deformation. Figure 14H,M indicates that the DG zone has higher KAMs and larger SSBs and LAGBs than the BM because of induced strain and generated sub-boundaries. Contrary to this area, lower KAMs and larger HAGBs in the FG area in Figure 14G,L were consistent with the generation of new grains with consuming dislocation.

Results for sample B suggest that DP microstructures with non-deformed fine grains of 1.4 µm will generate when the temperature reaches higher than A_c1_. The grain size should be determined by the balance of dynamic recrystallization, phase transformation, and diffusive grain coarsening. The martensite region was initially austenitized and new grains were generated with dynamic recrystallization while new ferrite grains also generated along the grains. The larger grain size than FG in sample A suggests that grain coarsening was relatively dominant over new grain generation in sample B, although the quantification of their balance is difficult. With increasing temperature over Ac_3_, grain coarsening became more dominant and phase transformation to martensite determined microstructures in this study. Grain size (average of 4.4 µm) was larger than that of the SZ in Aktarer et al.’s study [18] because the tool did not directly stir material, and material flow tended to be smaller in pinless FSSP than FSP.

### 3.4. Local Mechanical Properties for Low-Temperature Processing

As discussed in the previous section, the top surface of sample A showed a characteristic transition of the deformed microstructures at a low temperature. The local mechanical properties are essential to understand the phenomena at the initial stage of FSSP. Therefore, the authors performed nano-indentation on the top surface of sample A to determine the properties.

**Figure 16:**
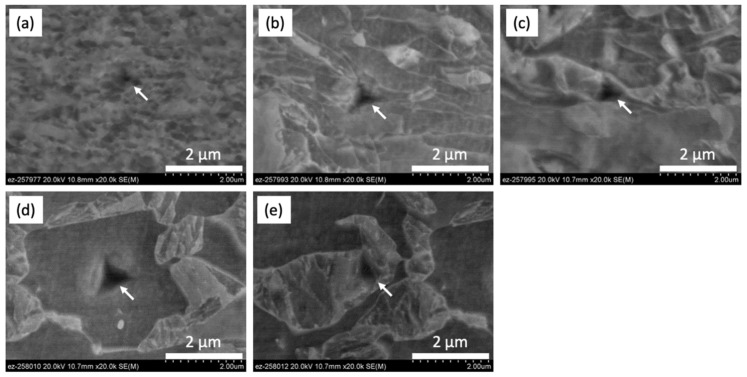
Representative SEM images of nano-indentation on each microstructure for sample A: (**a**) FG area; (**b**) ferrite; (**c**) martensite at the DG area; (**d**) ferrite; and (**e**) martensite at the BM. White arrow points indented location at each area.

Figure 16 shows SEM images of nano-indents on FG (a), ferrite (b), and martensite (c) at the DG zone; and ferrite (d) and martensite (e) at the BM. The indent on the FG area was across many grain or sub-grain boundaries as presented in Figure 16a. The nano-hardness of ferrite in DG area included the sub-grains while the indents on an MI were found as shown in Figure 16b. At the BM, the nano-hardness of ferrite and martensite was also measured as shown in Figure 16d,e.

**Figure 17:**
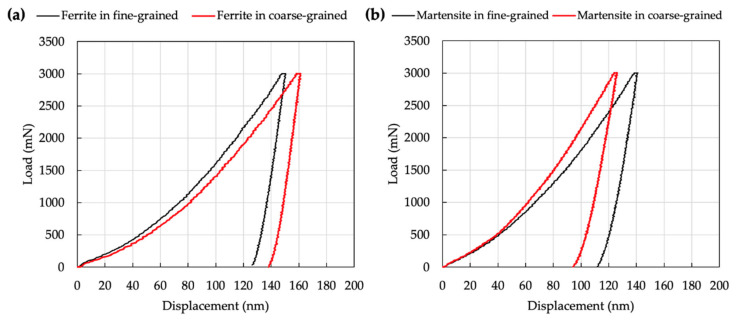
Representative load displacement curve of FG and DG areas for sample A: (**a**) ferrite and (**b**) martensite.

Figure 17 shows representative load displacement curves on ferrite (a) and martensite (b) during loading and unloading of nano-indentations on sample A. Although applied peak load was the same for each condition, the final indentation depth at which the applied loads become zero on unloading were different. Overall, a longer final indentation depth (130–140 nm) was found for ferrite, whereas a shorter final indentation depth (90–110 nm) was measured for martensite. This is because ferrite is softer than the martensite. The load displacement curves of ferrite and martensite in Figure 17 show a continuous increase without any pop-in behavior, which is a jump in displacement of the indenter associated with large-scale dislocation nucleation at the onset of plastic deformation. It is believed that carbide precipitation and recovery of matrix grains are limited in low-temperature FSSP. Hernandez et al. showed continuous load displacement curves without pop-in behavior for both ferrite and martensite after resistance spot welding of DP steel, and suggested this was attributed to the low rate of carbide precipitation and incomplete recovery due to the rapid thermal cycles of heating and cooling [24].

**Figure 18:**
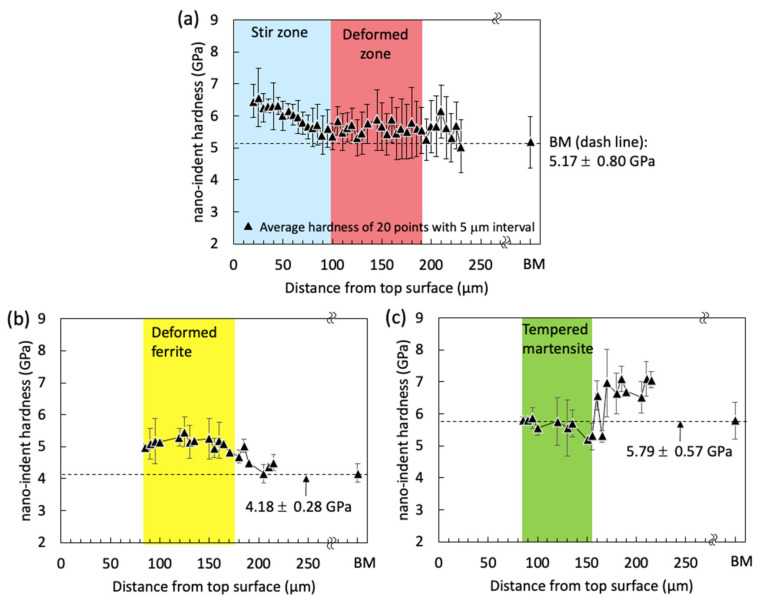
Nano-hardness distribution through thickness direction from the top surface for sample A: (**a**) nano-hardness profiles showing two distinctive regions (SZ and deformed zone in previous Figure 6a). Each point was the average of 20 measurements with standard deviation; (**b**) ferrite only in Figure 18a; and (**c**) martensite only in Figure 18a. The averaged nano-hardness value for ferrite and martensite in the BM is indicated by dash line in each figure.

Figure 18a plots averaged 20 nano-hardness indentations with standard deviation from the top surface to bottom surface for sample A. This averaged nano-hardness could include effects of different microstructures and grain sizes based on the different thermal and mechanical effects. Overall, the nano-hardness profile tended to increase as the nano-indentations came closer to the top surface as shown in Figure 18a. The averaged nano-hardness was 6.01 ± 0.61 GPa in the SZ and 5.61 ± 0.73 GPa in the deformed zone, while averaged nano-hardness for the base metal was 5.17 ± 0.80 GPa. The average nano-hardness distribution shown in Figure 18a presents a similar trend to the measured Vickers microhardness in previous Figure 5a. To distinguish nano-hardness for individual ferrite and martensite only, the nano-hardness distribution for each microstructure was separately presented in Figure 18b,c. Because differentiating ferrite and martensite near the top surface was difficult because of grain refinement in the SZ, the nano-hardness profile for each microstructure was plotted approximately 100 µm away from the top surface (i.e., the deformed zone in Figure 6). As presented in Figure 18b, the averaged nano-hardness value of ferrite was increased as closed to the top surface. Based on SEM, EBSD, and nano-indentation measurements, the averaged nano-hardness values of deformed and non-deformed ferrite were found to be 5.13 ± 0.41 GPa at a distance from 85 to 170 µm and 4.36 ± 0.28 GPa at approximately 200 µm away from the top surface, respectively. The averaged nano-hardness value for ferrite in the BM was measured to be 4.17 ± 0.28 GPa. 22.7% of the nano-hardness value was increased for ferrite at regions by FSSP compared with the ferrite in the BM. Delincé et al. reported that nano-hardness for ferrite in dual-phase steel increases from 2.57 ± 0.05 GPa to 3.29 ± 0.46 GPa, by almost 28.0%, with refining the grain size from 3.9 to 0.7 µm because of the grain boundary strengthening effect [31]. Although the chemical composition was different from their investigation, the nano-hardness results shown in Figure 18 suggest that the dominant factor on local mechanical properties at the mechanically stirred FG area (i.e., near the top surface) is grain boundary strengthening. In the FG zone, many grain boundaries were inevitably involved during FSSP. Nano-hardness values of the martensite were 5.66 ± 0.50 GPa at 100 µm away and 6.90 ± 0.51 GPa at 200 µm away from the top surface, while averaged nano-hardness for martensite in the BM was 5.79 ± 0.57 GPa as seen in Figure 18c. The measured nano-hardness value for martensite at 100 µm away from the top surface was slightly lower than the value of martensite in the BM. The martensite at this location was slightly tempered because frictional heat was generated during FSSP. Similarly, reduction of nano-hardness was observed when martensite was annealed [32].

### 3.5. Strengthening Effect in Processed Area

For further understanding of the dominant factor of local mechanical properties in the FSSPed area, the authors estimated and compared the local yield stress in samples A, B, and C as well as the BM by calculation from the Vickers microhardness and accumulation of the strengthening effect. The authors selected representative areas such as the FG and DG zones because of the thermomechanical effects from FSSP. First, SEM images and the characteristics of each evaluated area were correlated with Vickers microhardness in Figure 5. The measured Vickers microhardness for the FG zone in sample A was 530 Hv (in Figure 5a) and the f_M_ was 0.60. The measured Vickers microhardness for the DG zone in sample A was 476 Hv in Figure 5a and the f_M_ was 0.60. Sample B had a Vickers microhardness of 412 Hv as noted in Figure 5b and an f_M_ of 0.58. Sample C showed fully martensite structures and a Vickers microhardness of 430 Hv as noted in Figure 5c and an f_M_ of 1.0. The BM had a Vickers microhardness of 328 Hv and an f_M_ of 0.60.

The Vickers microhardness can be correlated to the yield stress. Pavilina and Van Tyne found a good relationship between yield stress and Vickers microhardness for steels [23].

(1) $$\sigma_{y}=-94.8+2.466\times \mathrm{Hv}$$

where Hv is the Vickers microhardness. For this reason, Equation (1) was used to calculate the yield stress of the BM. The yield stress of the BM was estimated to be 714 MPa, which is close to the average yield stress of 690 MPa from the literature [32]. Next, the yield stresses of the FG zone and the DG zone near the top surface in sample A were calculated to be 1212 and 1079 MPa, based on the measured Vickers microhardness values. In the same manner, the yield stresses of sample B and sample C were estimated to be 921 and 1146 MPa, respectively. Calculated yield stress for samples A, B, and C were higher than the value of the BM because of thermomechanical effects from FSSP. Table 2 summarizes the measured Vickers microhardness, calculated yield stress, and f_M_ for the BM, sample A, B, and C.

Further analysis was made to study each strengthening effect for FSSPed steels. Several investigations have been conducted to estimate the dominant factor on the mechanical properties of thermomechanical processing, such as FSW [19,20,21,22,33] for steels. Mazaheri et al. estimated the yield stress of ultra-FG DP steel by accumulating each strengthening effect [22,34]. Baker et al. reported the attribution of change in the particle and dislocation strengthening mechanism by comparing the stress value of the BM and Hall-Petch line of the FSWed oxide dispersion strengthened steel [19]. Li et al. explained the difference of the work hardening capacity of FSWed reduced-activation ferritic/martensitic steel by comparing the Hall-Petch relationship and dislocation strengthening [20]. The estimation of each strengthening effect is essential to understand the effect on mechanical properties.

In this work, yield stress in the FSSPed zone was estimated from the Vickers microhardness and microstructure characterizations in the previous sections to reveal the dominant strengthening factor. The yield strength of AHSS can be estimated as following equation [22]:

(2) $$\sigma_{y}=\sigma_{o}+\sigma_{s}+\sigma_{g}+\sigma_{d}$$

where *σ_0_*, *σ_s_*, *σ_g_*, and *σ_d_* are the strengthening effects caused by lattice friction stress, solid solution, grain refinement, and dislocation, respectively. Individual strengthening effect was calculated in the following equations.

The Peierls-Nabarro friction stress, *σ_0_*, and dislocation strengthening, *σ_d_*, are as following:

(3) $$\sigma_{0}=\left(\frac{2G}{1-\nu }\right)exp\left(-\frac{2\pi w}{b}\right)$$

(4) $$\sigma_{d}=\alpha Gb\rho^{\frac{1}{2}}$$

where *b* is the Burger’s vector, *G* is the shear modulus, *ν* is the Poisson’s ratio, *w* is the dislocation width, α is the material constant with a value of 0.33, *M* is the Taylor factor, *G* is the shear modulus, and *σ* is the dislocation density. For Equation (3), the values for *G*, *b*, and *ρ* are 80,000 MPa, 2.5 × 10^–10^ m, and 2.75 × 10^13^ m^–2^, respectively. The authors used the constant value of *σ_0_* of 50 MPa for the poly-crystalline pure iron at room temperature as well as in Mazaheri et al.’s work [22]. The value for *σ_d_* was calculated to be 103.8 MPa. The values related to dislocation should be deviated at different locations because of different thermal and mechanical effects from various processing conditions. Therefore, the authors calculated the strengthening effect based on the BM and increase in grain refinement hardening effect under an assumption that the other strengthening effects were not changed.

The solution strengthening effect, *σ_s_*, of the ferrite grain was estimated by the following:

(5) $$\sigma_{s}=4570\;\left(\%C\right)+83\;\left(\%\mathrm{Si}\right)+37\;\left(\%\mathrm{Mn}\right)+60\;\left(\%\mathrm{Cr}\right)$$

Carbon content in ferrite grain is assumed to be 0.015%, also referred in [22]. The solution strengthening effect was calculated to be 221 MPa.

The grain refinement hardening effect was estimated by the Hall-Petch equation:

(6) $$\sigma_{g}=k_{y}d^{-\frac{1}{2}}$$

where *d* is the average ferrite grain size and *k_y_* is the constant. The *k_y_* of HSLA steel, 0.55 MPa m^1/2^, was assumed in this study. The *σ_g_* for the BM was 352 MPa, and the *σ_g_* for FG and DG zones in sample A was calculated to be 710 and 434 MPa, respectively. The *σ_g_* for sample B was calculated to be 464 MPa. Summing up all the calculated values from Equations (3)–(6) led to the local yield stress (k_y_) as following: BM = 620 MPa; FG of sample A = 1084 MPa; DG of sample A = 809 MPa; sample B = 839 MPa; and sample C = 636 MPa.

**Figure 19:**
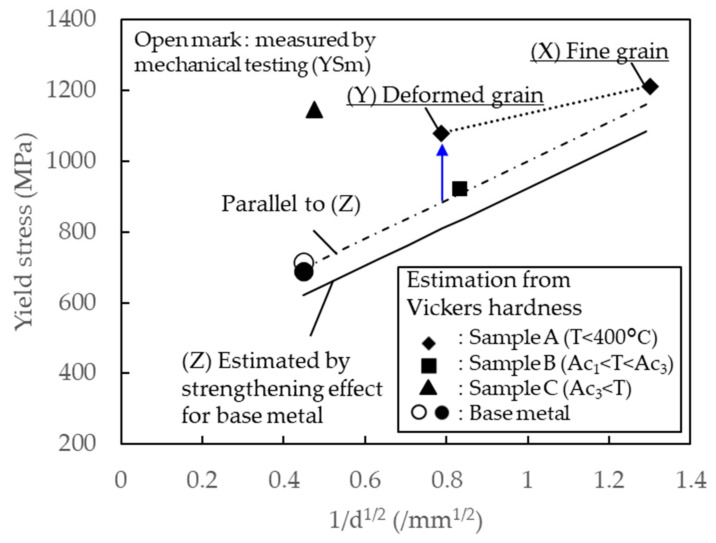
Summary of the local yield stress estimated by the local Vickers microhardness for the BM and samples A, B, and C.

Figure 19 summarizes the calculated yield stress based on strengthening factors with respect to the inverse of measured grain size. The calculated k_y_ (solid line) was lower than the yield stress by mechanical test (YS_m,_) (open circle in Figure 19) for several reasons, especially higher dislocation density and higher constants in the dislocation strengthening effect than the assumption of parameters in Equation (3). Kadkhodapour et al. a demonstrated dislocation density at a border of ferrite and martensite of dual-phase steel of 2.5 × 10^14^ m^−2^, 10 times higher than inside ferrite [35]. This inhomogeneous microstructure causes the lower estimation of *σ_d_* and *σ_0_*, However, the direct measurement is difficult because of limitations of the EBSD technique. Therefore, a dash-dot line was added to be started from YS_m_ with the same slope of the calculated yield stress (solid line) for the BM in this study. Additionally, the yield stress estimated from the Vickers microhardness for sample C was much higher than the calculation based on the strengthening effect. The f_M_ in sample C was 1.0, which is completely different from the BM, sample A, and sample B, whose fractions were around 0.6. Increasing the f_M_ causes high friction stress *σ_0_* in Equation (3) [36], which led to the large difference between the yield stress and the estimation based on the grain size. In this study, sample A, sample B, and the BM were compared because they had almost the same f_M_, and the grain size effect can be compared without the effect of friction stress. The calculated yield stresses in the FG zone of sample A and B were closely located on the dash-dot line, but the yield stress in the DG zone for sample A was clearly higher than the dash-dot line. This variation on the DG can be caused by the high dislocation density as previously explained by the KAM map in Figure 11. Through dynamic recrystallization, the dislocation density inside the grains reduced, and the reduction led to a lower yield stress. In sample B, the phase transformation redistributed the dislocation inside the grains, and the yield stress was estimated from the grain size effect and the BM with the same DP microstructures. Therefore, the dominant factor on the local mechanical properties can be confirmed as following: the effect of the grain boundary strengthening was dominant when severe grain refinement occurred in low-temperature FSSP. The neighboring area with severe deformation was largely affected by the induced strain, which was consistent with the dislocation density.

## 4. Conclusions

Pinless FSSP under different peak temperatures was performed using DP980 to study microstructure evolutions and their local mechanical properties. Low-temperature FSSP under 400 °C (sample A) generated a layer with a fine grain size of 0.6 µm or smaller. The local hardness of this layer was higher than 500 Hv, which is most affected by the grain boundary strengthening effect. Grain refinement should be driven by dynamic recrystallization due to severe deformation and high dislocation during FSSP. The dominant strengthening effect at the transient location between the fine grain layer and deformed microstructures changed from grain boundary strengthening to the dislocation. FSSP at the peak temperature between Ac_1_ and Ac_3_ (inter-critical) (sample B) formed a refined dual-phase (ferrite and martensite) structure with an average grain size of 1.4 µm. Phase transformation appeared to be dominant on the grain refinement even though dynamic recrystallization occurred. The average grain size of 4 µm with martensitic microstructure was obtained at a higher peak temperature (>Ac_1_) because of phase transformation from heat and cooling during FSSP.

## Figures and Tables

**Table 1:** Summary of down-selected FSSP conditions for different peak temperature and different microstructures.

Sample	Rotational Speed (rpm)	Plunging Force (N)	Dwell Time (s)
A	320	7100	7.1
B	500	6100	5.8
C	1800	5400	4.7

**Table 2:** Summary of Vickers microhardness, calculated yield stress based on Equation (1) and f_M_ for the BM and samples A (including FG, and DG zones), B, and C.

Sample	Vickers Microhardness (Hv)	Calculated Yield Stress (MPa) Based on Equation (1)	f_M_
BM	328	714	0.6
FG in A	530	1212	0.6
DG in A	476	1079	0.6
B	412	921	0.58
C	430	1146	1.0
